# Transmissible Endoplasmic Reticulum Stress: A Novel Perspective on Tumor Immunity

**DOI:** 10.3389/fcell.2020.00846

**Published:** 2020-10-07

**Authors:** Zhou Jiang, Geru Zhang, Liwei Huang, Yihang Yuan, Chenzhou Wu, Yi Li

**Affiliations:** State Key Laboratory of Oral Diseases, National Clinical Research Center for Oral Diseases, Department of Head and Neck Oncology, West China Hospital of Stomatology, Sichuan University, Chengdu, China

**Keywords:** cancer, tumor-derived extracellular vesicles, tumor immunity, unfolded protein response, transmissible ER stress

## Abstract

As the first compartment of the protein secretory pathway, the endoplasmic reticulum (ER) acts as a protein synthesis factory, maintaining proteostasis and ER homeostasis. However, a variety of intrinsic and extrinsic perturbations, such as cancer, can disrupt the homeostasis and result in a large accumulation of misfolded/unfolded proteins in the ER lumen, thereby provoking a specific cellular state addressed as “ER stress”. Then the unfolded protein response (UPR), an adaptive signaling pathway, is triggered to address the stress and restore the homeostasis. A novel aspect of ER stress is that it can be transmitted from cancer cells to tumor-infiltrating myeloid cells through certain cancer cell-released soluble factors, which is termed as transmissible ER stress (TERS) or ER stress resonance (ERSR). In this review, we provide a comprehensive overview of the link between cancer and ER stress as well as the possible soluble factors mediating TERS. We further elaborate the cell-extrinsic effects of TERS on tumor immunity, and how it indirectly modulates cancer development and progression, which is expected to add a new dimension to anticancer therapy.

## Introduction

The endoplasmic reticulum (ER) is an intracellular membranous organelle. As the first compartment of the protein secretory pathway, the ER acts as a protein synthesis factory. It is involved in the production, folding, modification, maturation, quality control and degradation of approximately one-third of all cellular proteins, and makes certain that only properly folded proteins can be transported to their intracellular or extracellular sites of action (Braakman and Bulleid, 2011; Stefan et al., 2011). Hence, the ER is closely associated with the maintenance of proteostasis and cellular homeostasis. However, a specific cellular state called “ER stress” will be triggered when ER homeostasis is disrupted by various intrinsic or extrinsic perturbations, including cancer (Papaioannou and Chevet, 2018), obesity (Hetz et al., 2020), neurodegeneration (Riaz et al., 2020), Alzheimer’s disease (Uddin et al., 2020), diabetes (Brooks-Worrell and Palmer, 2019), inflammation (Li et al., 2020), reactive oxygen species (ROS) production (Ochoa et al., 2018), etc.

In response to ER stress, the unfolded protein response (UPR) then will be triggered to overcome the stress and restore proteostasis and ER homeostasis by transcriptionally and translationally decreasing protein synthesis, increasing ER protein folding capacity and degrading the misfolded/unfolded proteins (Hetz, 2012). As an adaptive signaling pathway, the UPR is predominantly controlled by three transmembrane ER stress sensors: activating transcription factor 6 (ATF6), inositol-requiring enzyme 1 (IRE1) and protein kinase RNA-like ER kinase (PERK) (Zheng et al., 2016). The ER luminal domains of all the three sensors normally bind to the ER-resident chaperone, binding immunoglobulin protein (BiP), also known as glucose-regulated protein 78 (GRP78), locking them in monomeric, inactive states (Bertolotti et al., 2000; Shen et al., 2002). The three sensors will be released and activated when accumulational misfolded/unfolded proteins in the ER lumen competitively engage BiP (Pincus et al., 2010; Kopp et al., 2019). Then active ATF6 will translocate to the Golgi apparatus, while active IRE1 and PERK will subsequently activate downstream signaling cascades, driving mutually reinforcing signaling pathways for a common purpose: to initiate corrective measures to reestablish protein homeostasis and promote cell survival (Ye et al., 2000; Shen et al., 2002; Hetz, 2012; Sano and Reed, 2013; Frakes and Dillin, 2017). Nevertheless, if the UPR fails to get rid of the stress, then the UPR signals may switch from pro-survival to pro-death, including apoptosis, necroptosis and autophagic cell death (Sano and Reed, 2013; Hetz and Papa, 2018; Kim and Kim, 2018; Almanza et al., 2019).

## Cancer and ER Stress

As ER stress and the UPR have been reported in many kinds of cancers, it is widely acknowledged that cancer is one of the intrinsic ER perturbations that can result in ER stress as well as constitutive activation of the UPR signaling pathways (Hanahan and Weinberg, 2011; Wang and Kaufman, 2014; Kaneko et al., 2017). ER stress within cancer cells is initiated and amplified through multiple cell-intrinsic and cell-extrinsic mechanisms. First of all, high genetic instability and numerous non-synonymous mutations of cancers such as melanoma (Piwocka et al., 2006) and lung cancers (Volmer et al., 2013) can straightly destroy the folding capacity of proteins. Besides, high protein demand of uncontrollably and rapidly growing cancer cells can aggravate the burden of ER protein folding capacity, especially for some highly secretory cancers (Obeng et al., 2006; Holderfield et al., 2014). Furthermore, hypoxia, starvation, and lactic acidosis resulting from the depletion of oxygen and nutrients in the tumor microenvironment (TME) can also fuel ER stress (Vaupel et al., 1989; Giampietri et al., 2015; Chipurupalli et al., 2019; Xia et al., 2019). Notably, it is reported that several anticancer drugs can bring about ER stress *in vitro*, whose effects *in vivo* are unclear yet (Ma et al., 2014; Jeon et al., 2015; Pozzi et al., 2016).

In fact, there is an intimate interaction between cancer and ER stress, in other words, cancer can result in ER stress, which in turn can alter cancer development and progression. First of all, if cells can successfully impose restrictions on pro-apoptotic UPR outputs, IRE1 and PERK signaling pathways will facilitate the survival and growth of cancer cells under hypoxia and nutrient deprivation *in vivo* (Koumenis et al., 2002; Romero-Ramirez et al., 2004; Chen et al., 2014). Moreover, inducing intracellular autophagy can also sustain the survival of cancer cells. It is demonstrated that PERK-mediated autophagy is required to resist anoikis, a type of cell death as a result of extracellular matrix (ECM) detachment (Avivar-Valderas et al., 2011; Dey et al., 2015). Secondly, PERK induces epithelial-to-mesenchymal transition (EMT), a significant biological process for epithelial-derived malignant tumor cells to acquire the ability to migrate, invade and form tumorsphere, by silencing E-cadherin or overexpressing Twist, which was verified to be inhibited by a small-molecule PERK inhibitor (Feng et al., 2014; Dey et al., 2015). In addition, it is suggested that the PERK/eIF2α arm of the UPR enhances migration and invasion through induction of metastasis-associated LAMP3 *in vivo* and *in vitro* (Mujcic et al., 2013). Furthermore, multiple branches of the UPR are found contributive to a p38-dependent program of anti-proliferative dormancy of cancer cells during metastasis, which insulates these disseminated cells from adverse microenvironmental conditions such as hypoxia, glucose deprivation and even many anticancer drugs that rely on active proliferation (Ranganathan et al., 2006; Schewe and Aguirre-Ghiso, 2008; Bartkowiak et al., 2015). Thirdly, the UPR signaling pathways provide rapidly growing solid cancers with essential vascularization, which is conducive to providing adequate oxygen and nutrients while removing harmful substances. PERK translationally upregulates the vessel growth and stabilization factors: type 1 collagen inducible protein (VCIP) and platelet-derived growth factor receptor β (PDGFRβ) (Blais et al., 2006). Similarly, IRE1/XBP1, PERK/ATF4 and ATF6 can transcriptionally upregulate vascular endothelial growth factor A (VEGFA) under hypoxia and glucose deprivation (Ghosh et al., 2010). IRE1 also maintains the production of a broad variety of pro-angiogenic cytokines in malignant glioma (Auf et al., 2010). At last, ER stress likely leads to eventual drug resistance, for several anticancer drugs can induce autophagy, through which the survival of cancer cells may be achieved (Sui et al., 2013). For instance, PERK and IRE1/JNK signaling pathways can sustain autophagy, and this way gives rise to sorafenib resistance in hepatocellular carcinoma cell lines (Ogata et al., 2006; Shi et al., 2011). To sum up, the UPR will facilitate the survival, metastatic capacity, angiogenesis and drug resistance of cancer cells if ER stress is addressed in time. On the other hand, the UPR signaling will switch from pro-survival to pro-death if severe and unresolved ER stress leads to a huge accumulation of the misfolded/unfolded proteins, which is far beyond ER’s capacity to adapt and self-regulate (Hetz et al., 2015).

As a consequence, ER stress imposes a bidirectional regulation effect on cancer development and progression: both tumor-supporting and tumor-suppressive roles. However, current molecular insights into the mechanisms that allow the UPR signaling switch from pro-survival to pro-death pathways are still insufficient.

## Transmissible Endoplasmic Reticulum Stress

**FIGURE 1:**
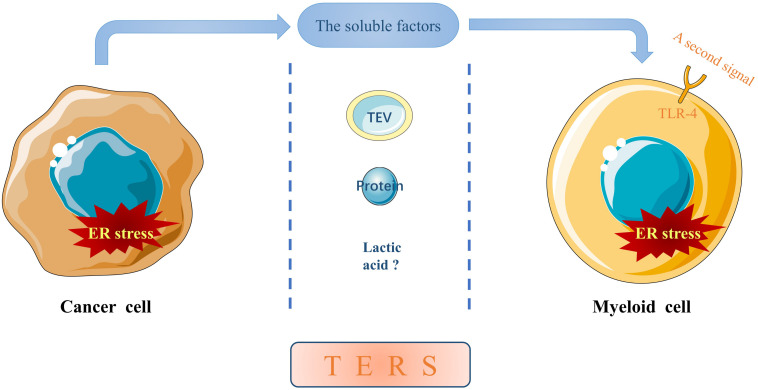
The soluble factors mediating TERS. ER stress can be transmitted from a primary ER stressed cancer cell to neighboring myeloid cells in the TME through certain cancer cell-released soluble factors, which may be TEVs, proteins or even lactic acid. In addition, ER stress in recipient myeloid cells can be potentiated by a second signal through TLR-4, although the molecule(s) remain elusive.

In addition to directly influencing the behaviors of cancer cells, ER stress is also involved in intercellular communication between tumor cells and non-tumor cells. Mahadevan et al. (2011) demonstrated for the first time that certain “soluble factors” released from prostate cancer cells undergoing ER stress can induce similar ER stress in bone marrow-derived myeloid cells, which is termed as transmissible ER stress (TERS). Subsequent studies further confirmed that TERS occurs in information communication between ER stressed cancers cells and other cancer cells or tumor-infiltrating immune cells such as tumor-associated macrophages (TAMs) (Urra et al., 2016; Rodvold et al., 2017). Hence, we propose a new concept of ER stress resonance (ERSR) to better understand the transfer of ER stress from a single primary cell to surrounding secondary cells through certain soluble factors. Inducing ER stress within these recipient cells can impede protective anti-tumor immunity while promoting survival and drug resistance in cancer cells and pro-inflammatory responses of immune cells, thus contributing to cancer development and progression (Mahadevan et al., 2012; Cullen et al., 2013; Cubillos-Ruiz et al., 2017; Rodvold et al., 2017; So, 2018; Di Conza and Ho, 2020). To date, limited evidence suggests that the soluble factors may be tumor-derived extracellular vesicles (TEVs), proteins or even lactic acid (Figure 1).

### The Soluble Factors: Tumor-Derived Extracellular Vesicles

It is now universally acknowledged that extracellular vesicles (EVs) can deliver some functional cargo to recipient cells, which plays a critical role in intercellular communication. Heusermann et al. (2016) discovered that about 90% EVs were delivered to the ER in recipient cells, and closely interacted with the ER membrane for 20 min, which may be conducive to cargo releasing. This encourages us to suppose that TEVs may directly deliver a great deal of non-specific cargo, such as misfolded/unfolded proteins to the ER in recipient cells and trigger ER stress. Beyond that, TEVs can also deliver some specific molecules. Javeed et al. (2015) demonstrated that pancreatic cancer caused paraneoplastic β-cell dysfunction by releasing adrenomedullin+ (AM+) exosomes into β-cell, which induced ER stress and inhibited insulin secretion due to the eventual failure of the UPR. Pancreatic cancer-released exosomes internalize and bind with endocytosed AM receptors (ADMRs), activating the cAMP-dependent signaling pathway. Subsequently, ER stress sensors are activated, featured with increased Bip/proinsulin coupling in the ER and overproduction of insulin owing to excessive exosomal AM. In addition, Wu et al. (2019) showed that urinary bladder cancer cell-derived EVs contained many proteins that could increase cell metabolism, which disrupted proteostasis and induced ER stress in recipient bladder epithelial cells. Of note, protein disulfide isomerase (PDI), an ER-resident protein, may be the key protein, since it takes charge of the formation of disulfide bonds of properly folded proteins. Interestingly, it is observed that the transformed recipient cells exhibit the abnormal accumulation of small-sized mitochondria coupled with the disordered ER. The downregulation of mitofusin 2 (Mfn2), a key protein in mitochondrial fusion and the bridge between mitochondria and the ER, has been shown to overactivate the UPR signaling pathways in recipient cells, since Mfn2 may be an upstream repressive modulator of PERK (De Brito and Scorrano, 2008; Ngoh et al., 2012; Muñoz et al., 2013). Therefore, it is of vital significance to investigate the potential link between cancer TEVs and Mfn2.

### The Soluble Factors: Proteins

In addition to TEVs, secretory proteins have also been suggested to be engaged in the molecular mechanisms of TERS from tumor cells to non-tumor cells. Wei et al. (2019) discovered that secreted Golgi protein 73 (GP73), an effective serum biomarker for hepatocellular carcinoma (HCC), was overexpressed and only secreted from abnormal HCC cells under ER stress both *in vivo* and *in vitro*, which was indispensable for the transfer of ER stress from hepatoma cells to macrophages. The possible mechanism is that secreted GP73 binds directly to Bip of neighboring macrophages both at the cell membrane and in cytosolic compartment, where C terminus (52–401 aa) of GP73 and N terminus (1–290 aa) of Bip were essential for the interaction and the following activation of ER stress sensors in recipient cells. Besides, Mahadevan et al. (2011) found that TERS was potentiated by a second signal through Toll-like receptor 4 (TLR4) on recipient macrophage cell membrane. Early studies have suggested that the molecule(s) that binds to TLR4 is heat resistant, and the binding may be potentiated by heat treatment (Mahadevan et al., 2011).

### The Soluble Factors: Lactic Acid?

A recent study demonstrated that murine tumor cells cultured under ER stress conditions produced lactic acid, a by-product of aerobic glycolysis, which induced the transcription of VEGF and arginase 1 in recipient macrophages (Colegio et al., 2014). It raises the possibility of that the soluble factor mediating TERS may be lactic acid, since the phenotype partially matches that of myeloid cells as the targets of TERS (described below). Nevertheless, Colegio et al. (2014) demonstrated that lactic acid promoted tumor growth through inducing VEGF expression and the M2-like polarization of TAMs rather than inducing TERS. Therefore, whether TERS is related to lactic acid still requires more clearer and stronger evidence.

## The Effects of TERS on Tumor Immunity

In the past few decades, the effects of ER stress on cancer cells have been described in detail, but the causes and effects of TERS from tumor cells to neighboring non-tumor cells are only beginning to be investigated. Here, we will briefly discuss the effects of TERS on tumor-infiltrating immune cells and inflammatory responses of these cells in the TME, as well as how it indirectly influences cancer development and progression.

### Immunosuppressive Effects

Oncogene mutations will facilitate antigen presentation, which stimulates an anti-tumor immunity to inhibit cancer development and progression. In order to attain the goal of immune escape, cancer cells are supposed to possess strong immunosuppressive capability in the TME that consists of stromal cells and infiltrating immune cells (Ramirez et al., 2019). TERS alter the development of anti-tumor immune responses by inducing ER stress and the UPR in tumor-infiltrating immune cells, including TAMs, bone marrow-derived dendritic cells (DCs), myeloid-derived suppressor cells (MDSCs) and T cells.

Transmissible ER stress can activate TAMs and induce a pro-inflammatory response (described below) in the TME (Mahadevan et al., 2011). The whole process not only markedly decreases the antigen processing and presenting capacity of DCs (possibly through the downregulation of tapasin) and the proliferation capacity of the cluster of differentiation 8^+^ (CD8^+^) T cells, but also provokes the overexpression of immunosuppressive molecules (Mahadevan et al., 2012). Of note, T cells are not the direct targets of TERS, indicating that the cell-extrinsic immunosuppressive effects of TERS are indirectly achieved through myeloid antigen-presenting cells which are the primary targets of TERS. For instance, DCs are especially sensitive to the UPR (Iwakoshi et al., 2007; Osorio et al., 2014). Zanetti et al. also demonstrated that macrophages and MDSCs as the primary targets of TERS might have a positive effect on tumor growth and metastasis *in vitro*, as evidenced by the upregulated expression of the UPR genes *BIP*, *CHOP* and *XBP-1S*, the increased production of inflammatory cytokines IL-6, IL-23 and tumor necrosis factor-α (TNF-α), and the enhanced expression of the immunosuppressive enzyme arginase 1 and the pro-angiogenic molecule VEGF (Zanetti et al., 2016).

**FIGURE 2:**
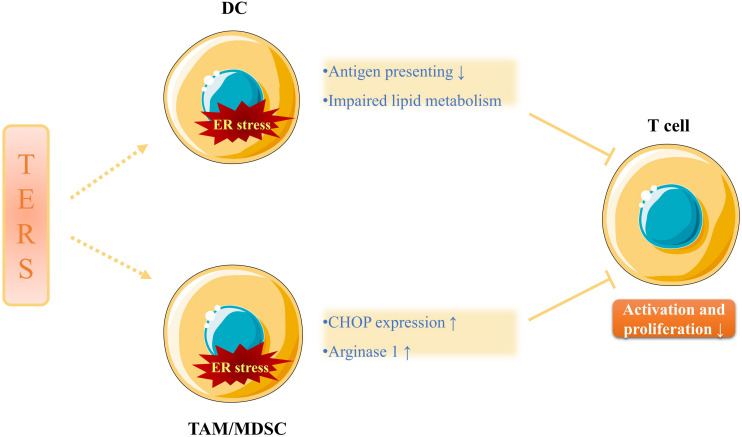
The immunosuppressive effects of TERS. TERS not only attenuates antigen presenting ability and impairs lipid metabolism of DCs, but also increases CHOP expression and arginase 1 production of TAMs/MDSCs, all of which inhibits the activation and proliferation of T cells. Therefore, anti-tumor immunity is undermined and cancer progression is promoted.

In addition, Cubillos-Ruiz et al. (2015) revealed that tumor-infiltrating DCs exhibited an increased expression of IRE1/XBP1, which accelerated the development and progression of ovarian carcinoma. In the same study, the tumor-infiltrating DCs also displayed the increased production of ROS that would disrupt ER homeostasis and lead to decreased anti-tumor immunity caused by impaired lipid metabolism and T cell function (Figure 2). Intriguingly, it was pointed out that certain soluble factors from the ascites of ovarian cancer patients lowered the expression of the glucose transporter 1 (GLUT1) on CD4^+^ T cells *ex vivo*, which imposed restrictions on their ability to make use of this essential nutrient (Song et al., 2018). Beyond that, Thevenot et al. (2014) disclosed that tumor-infiltrating MDSCs showed a high expression of PERK/CHOP in a mouse model, which was also observed in tumor-infiltrating macrophages *ex vivo*. These phenomena show great the significance for the cell-extrinsic effects of TERS on tumor immunity, since downregulated CHOP in tumor-infiltrating MDSCs is associated with declined T cell immune function (Thevenot et al., 2014). Similarly, Condamine et al. (2014) revealed that tumor-infiltrating MDSCs *ex vivo* exhibited upregulated expression of ER stress response genes, including *CHOP*, *XBP-1*, *BIP* and *ATF4*. However, the PERK/ATF4/CHOP axis will initiate cancer cell apoptosis if ER stress is excessively severe and unsettled (Condamine et al., 2014).

Taken together, all these studies have preliminary accounted for that TERS displays cell-extrinsic immunosuppressive effects in the TME and thus facilitates cancer development and progression, which subverts the body’s anti-tumor immunity (Mahadevan and Zanetti, 2011). Mild and solved ER stress can offer cancer cells and tumor-infiltrating immune cells in the TME with greater immunomodulatory capacity, whereas severe and lethal ER stress can trigger immunogenic cell death (ICD) and protective anti-tumor immunity (Pol et al., 2015). Hence, TERS and the UPR signaling pathways can be promising targets to either inhibit the cell-extrinsic immunosuppressive effects of TERS or to promote ER stress-induced apoptosis of cancer cells, which may pave the way for new strategies of anticancer immunotherapy and chemotherapy.

### Pro-inflammatory/Suppressive Effects

Previous studies have demonstrated that TERS-activated TAMs showed the increased production of cytokines, including IL-6, IL-23, and TNF-α, inducing a pro-inflammation response (Mahadevan et al., 2012; Zanetti et al., 2016). A recent study also revealed that nuclear factor kappa B (NF-κB), a regulator of downstream inflammatory signals of active IRE1 and PERK, was highly upregulated in ER stressed bladder cancer TEV-transformed cells. A few ER stress-associated inflammatory cytokines were found to be significantly upregulated, including leptin, chemokine CCL2 and transforming growth factor β (TGFβ), which confirmed the activation of inflammatory signals in TEV-transformed cells (Wu et al., 2019). In addition, Nakagawa et al. found that TERS seemed to accelerate obesity-driven hepatic tumorigenesis in a TNFα-dependent manner, suggesting that TERS might bring about cancer-promoting inflammation (Coussens et al., 2013; Nakagawa et al., 2014). The inflammatory environment within the TME is shown to be conducive to establishing a metastatic niche and inducing cancer cell “stemness,” which further accelerates cancer development and progression (Plaks et al., 2015).

However, the effects of TERS on recipient tumor-infiltrating immune cells are not completely pro-inflammation. It is also consistently observed that myeloid cells exhibited the elevated expression of immunosuppressive molecule arginase 1, which can inhibit the activation of T cells (Norian et al., 2009; Mahadevan and Zanetti, 2011). Therefore, as the targets of TERS, tumor-infiltrating myeloid cells show both pro-inflammatory and anti-inflammatory phenotype.

## Concluding Remarks and Future Perspectives

There is an intimate interaction between cancer and ER stress. Cancer is one of the intrinsic ER perturbations that can give rise to ER stress and the constitutive activation of the UPR signals. In return, ER stress imposes a bidirectional regulation effect on cancer development and progression: both tumor-supporting and tumor-suppressive roles. Specifically, the UPR promotes the survival, metastatic capacity, angiogenesis and drug resistance of cancer cells if ER stress is overcame in time, whereas severe and lethal ER stress induces cancer cell death. An intriguing question posed by the UPR signaling switching from pro-survival to pro-death is what the threshold between two distinct-different regulatory signals is. Further research is required to answer this question.

In addition to directly regulating cancer cell phenotype, ER stress can be transmitted from cancer cells to neighboring tumor-infiltrating immune cells within the TME through certain cancer cell-released soluble factors, resulting in TERS among the two types of cells. Currently, limited evidence suggests the soluble factors may be certain functional cargo in TEVs, proteins or even lactic acid. Of note, TERS is found to play an immunosuppressive role in tumor-infiltrating immune cells, and these cells display pro-inflammatory/suppressive responses, which indirectly facilitates cancer development and progression. Hence, it is of paramount importance to further elucidate molecular mechanisms of TERS, since the soluble factors mediating TERS may constitute key points to improve the efficacy of broad-spectrum anticancer therapies.

## Author Contributions

ZJ wrote the manuscript. GZ, LH, YY, and CW collected the related references and edited the manuscript. YL provided guidance and revised the manuscript. All authors approved the final manuscript.

## Conflict of Interest

The authors declare that the research was conducted in the absence of any commercial or financial relationships that could be construed as a potential conflict of interest.
